# Wild populations of malaria vectors can mate both inside and outside human dwellings

**DOI:** 10.1186/s13071-021-04989-8

**Published:** 2021-10-07

**Authors:** Ismail H. Nambunga, Betwel J. Msugupakulya, Emmanuel E. Hape, Issa H. Mshani, Najat F. Kahamba, Gustav Mkandawile, Daniel M. Mabula, Rukiyah M. Njalambaha, Emmanuel W. Kaindoa, Letus L. Muyaga, Marie R. G. Hermy, Frederic Tripet, Heather M. Ferguson, Halfan S. Ngowo, Fredros O. Okumu

**Affiliations:** 1 Environmental Health and Ecological Sciences Department, Ifakara Health Institute, P.O. Box 53, Ifakara, Tanzania; 2 Institute of Biodiversity, Animal Health and Comparative Medicine, University of Glasgow, Glasgow, UK; 3 School of Life Science and Bioengineering, The Nelson Mandela African Institution of Sciences & Technology, Arusha, Tanzania; 4 School of Public Health, Faculty of Health Sciences, University of the Witwatersrand, Park Town, Republic of South Africa; 5 Disease Vector Group, Chemical Ecology, Department of Plant Protection Biology, Swedish University of Agricultural Sciences, Alnarp, Sweden; 6 Centre for Applied Entomology and Parasitology, School of Life Sciences, Keele University, Newcastle-under-Lyme, UK

**Keywords:** Mosquito mating, *Anopheles funestus*, *Anopheles arabiensis*, Eurygamic species, Malaria, Tanzania

## Abstract

**Background:**

Wild populations of *Anopheles* mosquitoes are generally thought to mate outdoors in swarms, although once colonized, they also mate readily inside laboratory cages. This study investigated whether the malaria vectors *Anopheles funestus* and *Anopheles arabiensis* can also naturally mate inside human dwellings.

**Method:**

Mosquitoes were sampled from three volunteer-occupied experimental huts in a rural Tanzanian village at 6:00 p.m. each evening, after which the huts were completely sealed and sampling was repeated at 11:00 p.m and 6 a.m. the next morning to compare the proportions of inseminated females. Similarly timed collections were done inside local unsealed village houses. Lastly, wild-caught larvae and pupae were introduced inside or outside experimental huts constructed inside two semi-field screened chambers. The huts were then sealed and fitted with exit traps, allowing mosquito egress but not entry. Mating was assessed in subsequent days by sampling and dissecting emergent adults caught indoors, outdoors and in exit traps.

**Results:**

Proportions of inseminated females inside the experimental huts in the village increased from approximately  60% at 6 p.m. to approximately 90% the following morning despite no new mosquitoes entering the huts after 6 p.m. Insemination in the local homes increased from approximately 78% to approximately 93% over the same time points. In the semi-field observations of wild-caught captive mosquitoes, the proportions of inseminated *An. funestus* were 20.9% (95% confidence interval [CI]: ± 2.8) outdoors, 25.2% (95% CI: ± 3.4) indoors and 16.8% (± 8.3) in exit traps, while the proportions of inseminated *An. arabiensis* were 42.3% (95% CI: ± 5.5) outdoors, 47.4% (95% CI: ± 4.7) indoors and 37.1% (CI: ± 6.8) in exit traps.

**Conclusion:**

Wild populations of *An. funestus* and *An. arabiensis* in these study villages can mate both inside and outside human dwellings. Most of the mating clearly happens before the mosquitoes enter houses, but additional mating happens indoors. The ecological significance of such indoor mating remains to be determined. The observed insemination inside the experimental huts fitted with exit traps and in the unsealed village houses suggests that the indoor mating happens voluntarily even under unrestricted egress. These findings may inspire improved vector control, such as by targeting males indoors, and potentially inform alternative methods for colonizing strongly eurygamic *Anopheles* species (e.g. *An. funestus*) inside laboratories or semi-field chambers.

**Graphical Abstract:**

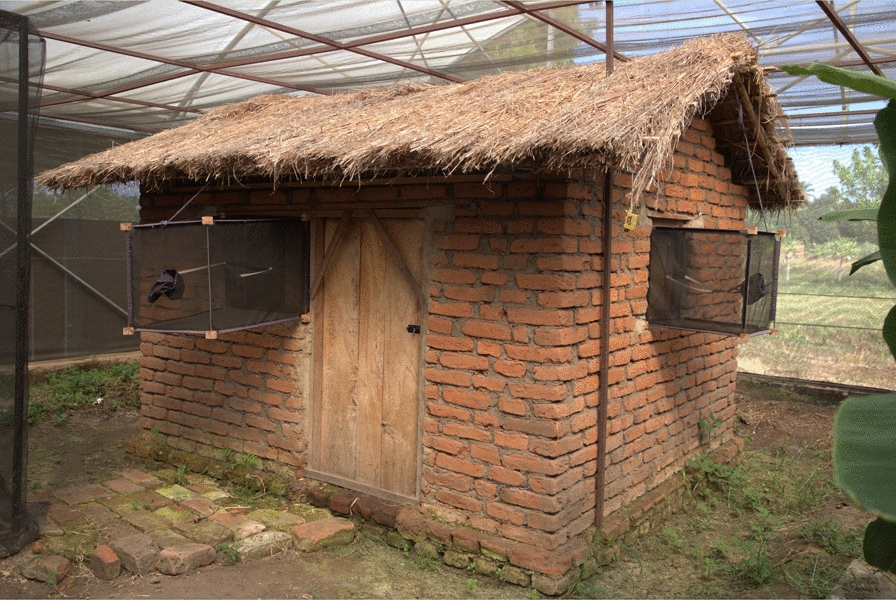

**Supplementary Information:**

The online version contains supplementary material available at 10.1186/s13071-021-04989-8.

## Background

Members of the *Anopheles gambiae* complex and *Anopheles funestus* group mediate the majority of malaria parasite infections in many parts of sub-Saharan Africa [1–4], where 94% of all malaria deaths and 93% of all cases occurred in 2019 [5]. Countries in this region rely mostly on the use of insecticide-treated nets (ITNs), indoor residual spraying (IRS), as well as effective case management and behavior change communication strategies to control malaria [6, 7]. These interventions have played a major role in reducing the burden of malaria in the past decades, with vector control interventions contributing to the majority of the success [8–11]. Unfortunately, recent evidence indicates that progress against malaria is plateauing and that gains may be lost [6].

While the major vector control tools, ITNs and IRS, primarily target mosquitoes that bite and rest indoors [12–15], it is widely accepted that additional tools targeting other aspects of the mosquito life-cycle will be important to further drive progress against malaria [16–18]. One of the opportunities for designing new approaches or optimizing current ones is through an improved understanding of the reproductive behaviors of mosquitoes. For example, a greater understanding of mosquito swarming and mating can enable improved targeting of male mosquitoes [19]. Technologies such as sterile insect techniques (SIT) [20], genetic modification of mosquitoes (GMM) [21] and space spraying of mosquito swarms [19, 22] are some of the interventions that exploit the mating behavior of mosquitoes. In Burkina Faso, space spraying of mosquito swarms reduced mosquito populations by up to 80% in the intervention village [19].

Both GMM and SIT have been explored to varying degrees for impact on different insect and pest populations [23–25]. Their successes can be greatly improved if the behaviors of both male and female insects are investigated and exploited. In addition, some of the complementary tools currently being evaluated, notably attractive targeted sugar baits (ATSBs) [26–28], could also be improved by possibly targeting both male and female mosquitoes inside and outside houses.

Like many other diptera, mosquito mating occurs mostly in swarms outside homes [29], where groups of male mosquitoes, usually of the same species, aggregate at specific times and places [30]. Several minutes after mosquito swarming begins, females join the swarm to seek a mate and leave in copula [22]. Mosquito swarming is thought to be mediated by the circadian rhythm of male mosquitoes [31, 32] and the presence of specific environmental markers in a particular locality [33, 34]. Recent evidence shows that this behavior might also be mediated by specific aggregation pheromones produced by male mosquitoes [35].

While *Anopheles* swarms often occur outdoors in open spaces at dusk [36–38], during recent collections of resting mosquitoes in south-eastern Tanzania, large numbers of male *Anopheles* were observed resting inside houses at dusk, at a time the males are supposedly in swarms (BJ Msugupakulya, unpublished). In a separate study, swarms of *An. funestus* were observed very close to human houses (often at the level of eaves) [36], unlike those of *An. arabiensis* which tended to occur mostly at the edge of the villages [33]. An experimental hut observation in west Africa found that the daily insemination rates were approximately 5% higher in exit traps than entry traps, implying that some limited mating could occur indoors independent of outdoor swarms [39].

In addition to advancing vector control, a greater understanding of *Anopheles* mating could also improve capabilities for colonizing some of the species that are otherwise difficult to rear inside laboratories. One particular example is *An. funestus*, which dominates malaria transmission in several east and southern Africa zones [3, 40–43], yet there have been only two successful laboratory colonies using field-collected material from Mozambique and Angola [44]. Recent evidence from attempted colonization has highlighted mating as one of the bottlenecks to colonization [45]. Studying the conditions of successful mating both outdoors in the wild and in captivity could improve rearing of these and other eurygamic mosquito species.

In the present study, we therefore, investigated indoor and outdoor insemination rates in major malaria vectors (*An. funestus* and *An. arabiensis*) under both field and semi-field conditions, to inform new opportunities for improving their control and also to improve efforts to colonize various *Anopheles* species for laboratory studies.

## Methods

### Study sites

**Fig. 1:**
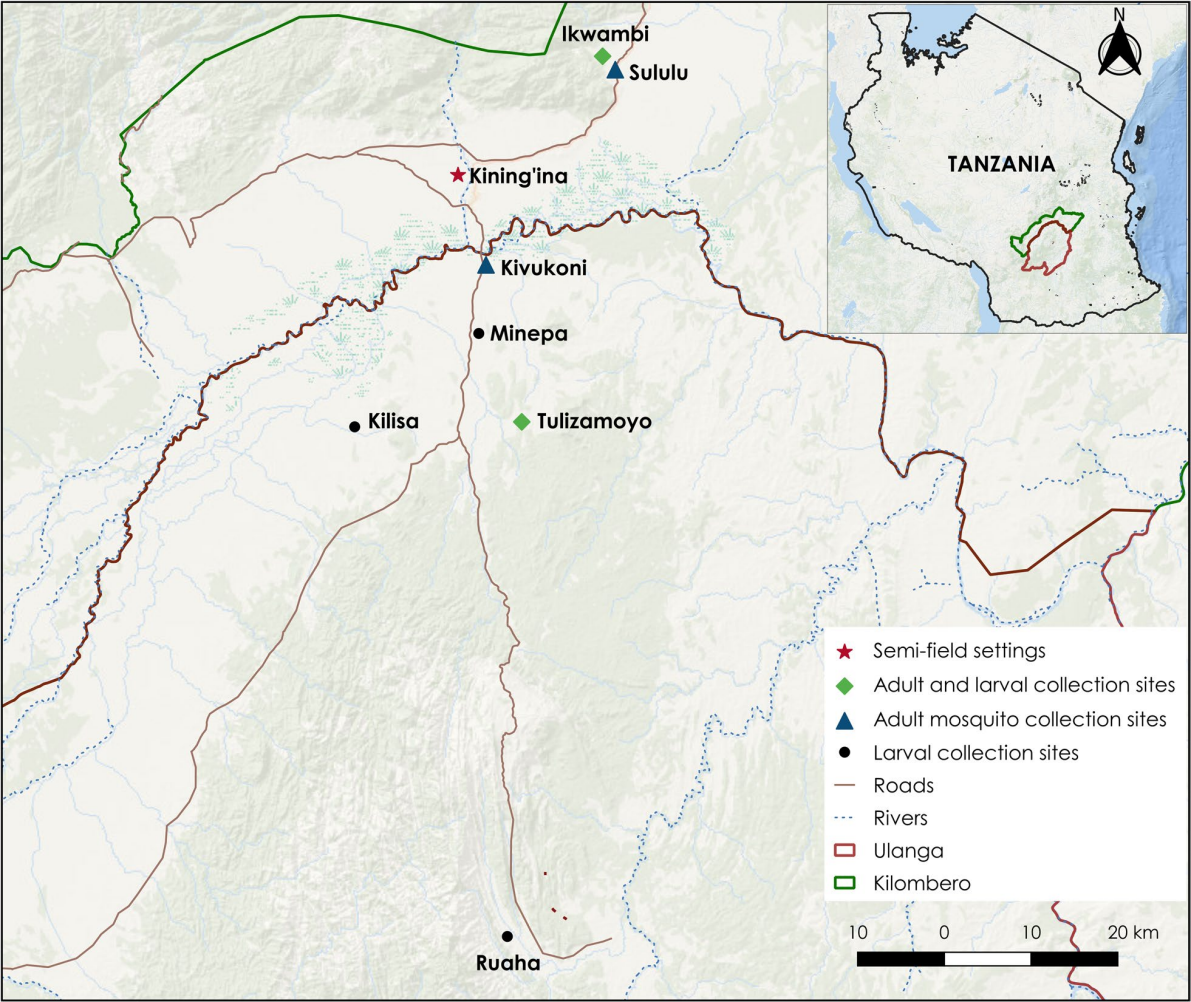
Map of Kilombero and Ulanga districts showing the locations where experiments were conducted

This study was implemented in four phases in the field and semi-field environments. The field studies were conducted in south-eastern Tanzania, in five villages located in Ulanga district, namely Kivukoni (– 8.2021**°**S, 36.6961°E), Minepa (– 8.1455°S, 36.4244°E), Tulizamoyo (− 8.3669°S, 36.7336°E), Kilisa (– 8.3721°S, 36.5584°E) and Ruaha (– 8.9068°S, 36.7185°E), and in two villages located in Kilombero district, Sululu (– 7.9973°S, 36.8317°E) and Ikwambi (– 7.9833°S, 36.8184°E) (Fig. 1). The principal malaria vectors in the area are *An. arabiensis,* and *An. funestus* [40, 41, 46, 47]. Several other anophelines and some culicine mosquito species are also present in the study area. The semi-field experiments were conducted inside large screen house compartments at the Mosquito City facility maintained by Ifakara Health Institute in Kining’ina village (– 8.1080°S, 36.6668°E) in Kilombero district. These semi-field systems were designed to mimic the natural environment, and have built-in experimental huts for controlled mosquito studies [48].

### Study procedures

The four study phases included: (i) assessment of indoor resting densities of male *Anopheles* mosquitoes relative to females in different house types; (ii) observations of insemination status of wild-caught female mosquitoes at different times of night inside volunteer-occupied experimental huts in the village; (iii) field observations to assess whether wild mosquitoes’ mate before or after entering local houses occupied by residents; and (iv) semi-field observations of wild-caught mosquitoes inside large screen house chambers to verify and quantify the insemination rates inside and outside experimental huts under controlled settings.

### Observations of male mosquitoes resting inside different types of houses

Mosquito collections were conducted in 80 houses in the four study villages (Fig. 1), targeting four common house types, namely: (i) 20 houses with thatched roofs and mud walls; (ii) 20 houses with thatched roofs and unplastered brick walls; (iii) 20 houses with metal roofs and unplastered brick walls; and (iv) 20 houses with metal roofs and plastered brick walls. Inside the houses, mosquitoes were collected from all potential resting surfaces using Prokopack aspirators (John W. Hock Company, Gainesville, FL, USA) to obtain total numbers of mosquitoes resting inside each house. Initially, mosquito collections were conducted only in the morning, separated into early morning (7–9 a.m.) and late morning (9–12 a.m.) collections, but additional collections were subsequently performed in the early evening (6–20 p.m.) and late at night (12 midnight–2 a.m.), as previously described by Msugupakulya et al.[49].

### Observations of insemination in wild mosquitoes caught inside volunteer-occupied experimental huts

**Fig. 2:**
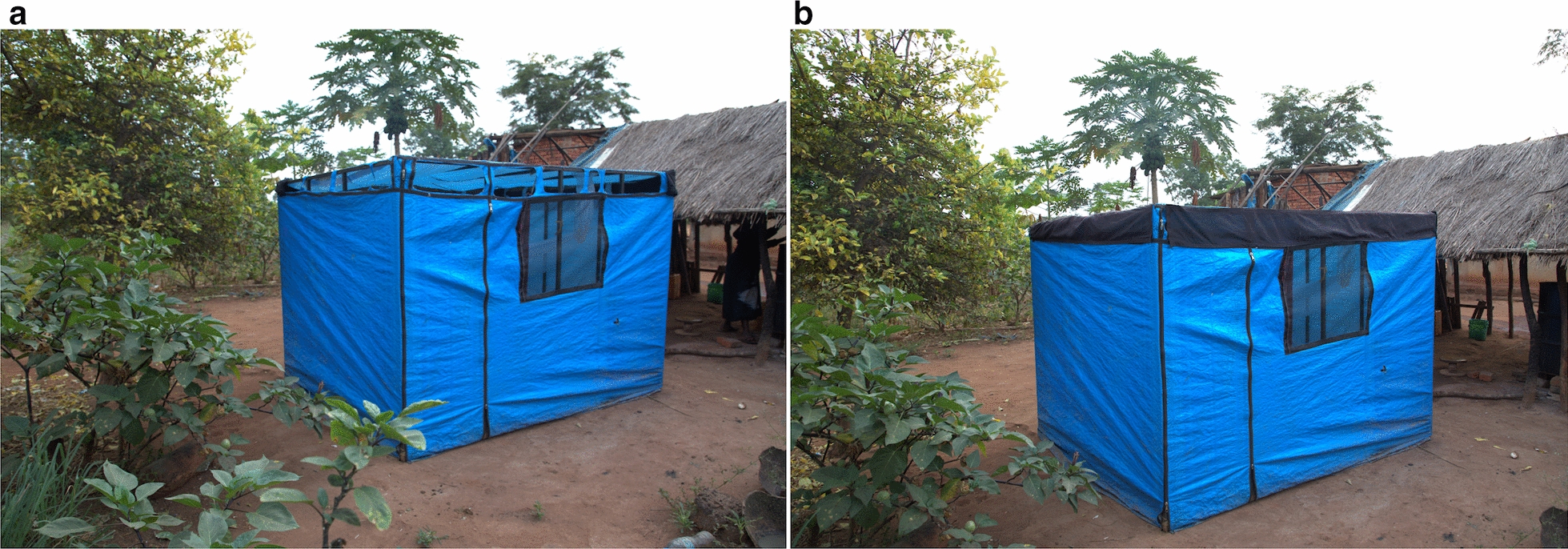
Experimental huts where mosquitoes were collected for assessment of insemination status of mosquitoes at different times points during the evening, night and following morning.** a** Eave spaces open to allow mosquito entry,** b** eave spaces closed for collection

This experiment was conducted in Tulizamoyo village using three volunteer-occupied experimental huts constructed for the study, as shown in Fig. 2. The same volunteers were used throughout the study, and each volunteer was allocated his own sleeping room (always the same room) for the duration of the study. These tent-styled huts consisted of easy-to-seal eave spaces and screened windows, and were located near other village houses used by residents (Fig. 2). During the study, the eave spaces remained open during the daytime (7 a.m. to 6 p.m.) to allow mosquito entry, and were closed at 6 p.m. Trained volunteers entered each experimental hut just before the huts were closed at 6 p.m. and collected mosquitoes using a Prokopack aspirator from multiple indoor surfaces for a total of 5 min each time. Without re-opening the huts after their closure at 6 p.m., follow-up collections were done at 11 p.m. and again at 6 a.m. the following morning, after which the huts were then re-opened. At each of these time points, the sampling was done at multiple locations inside the huts. In between collections, the volunteers slept under untreated nets inside the huts.

We hypothesized that the proportions of inseminated females would stay either similar (if no additional mating happened indoors after hut closures at 6 p.m.) or increase (if there was additional mating after 6 p.m.). Collected mosquitoes were kept in labeled cups and transferred to the laboratory for morphological identification and assessment of their insemination status.

The sampled female mosquitoes were initially identified using morphological keys for Afrotropical *Anopheles* mosquitoes [50, 51]. *Anopheles funestus* and *An. arabiensis* were dissected under a stereomicroscope. The seventh segment of immobilized mosquitoes was dissected to extract spermathecae, which were examined under a light microscope with a 10× magnification lens for insemination status. Female mosquitoes with filled long threads of coiled brown spermatheca were considered to be inseminated while those with clear and non-striated spermathecae were considered to be non-inseminated [52]. This study was conducted for 14 consecutive nights in the first round.

After the first round, the experiment was repeated for another 10 consecutive nights using the same procedures as described above, with the exception that all mosquitoes were immobilized by freezing in portable cooler boxes immediately after collection to avoid any possible mating that could happen inside the holding cups.

### Observation of insemination in wild female mosquitoes caught from local houses in the study village

An additional experiment to assess the insemination of malaria vectors was performed for 5 nights using natural houses where people live in the study village. Three thatched-roof and three iron roof houses, all of which were occupied by residents of the villages, were used for this experiment. During this phase of the experiment, the eaves were left open to allow mosquitos to freely enter and exit the dwellings. At 6 p.m. and 11 p.m. on the same evening and at 6 a.m. the following morning, mosquitoes were collected using Prokopack aspirators and immobilized immediately in the cooler box with ice packs to prevent any potential mating activity inside the holding cups and during transportation. These wild-caught mosquitoes were then transported to the insectary, and females were assessed for their insemination status as described above.

### Observations of insemination in wild-caught mosquitoes maintained under semi-field conditions

**Fig. 3:**
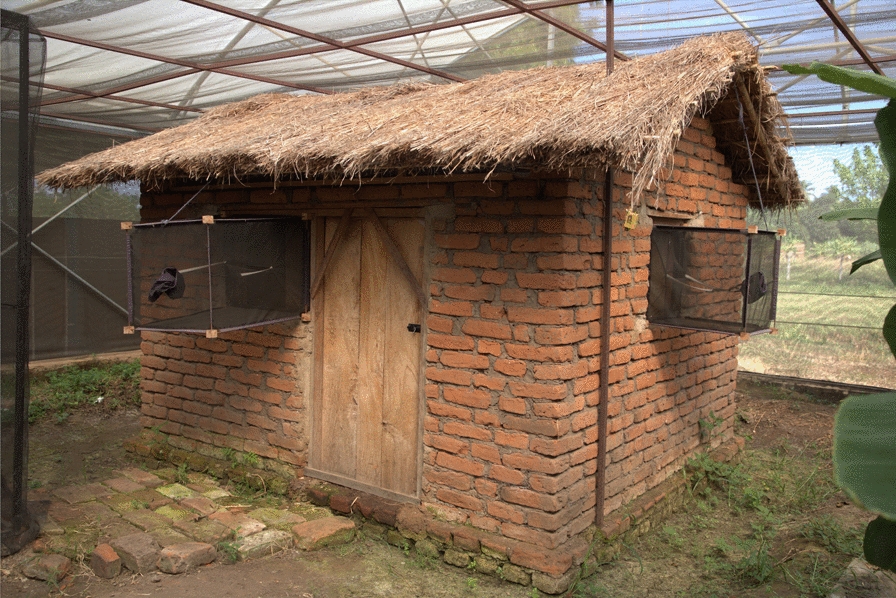
Experimental hut constructed inside the semi-field chambers and fitted with window exit traps

The semi-field system consisted of large multi-chambered screen houses with netting walls, enclosing village-like ecosystems of vegetation and water puddles [48]. Each chamber (9.6 × 9.6 m) had an experimental hut constructed to mimic the design of typical local houses used in rural Tanzania (Fig. 3). Three chambers were used for this study. The experimental huts in the selected chambers were completely sealed with mosquito netting on the eave spaces, but the windows were fitted with window exit traps to catch any mosquitoes attempting to exit the huts. With this design, mosquitoes inside the huts could attempt to exit (and be trapped in the window exit traps) but those outside could not enter the huts (since all eave openings were screened, doors were closed and windows were covered with the exit traps). For additional control, the doors were fitted with overlapping net curtains to prevent mosquitoes from flying in or out whenever someone entered.

Field collections of third and fourth instar *Anopheles* larvae were performed using a combination of standard 350-ml dippers (for small habitats) and 10-L buckets (for large habitats) to maximize densities, as previously described by Nambunga et al. [53]. The larvae were sorted, and only *Anopheles* larvae were used for further observations. The larvae were maintained in rearing basins and fed daily on TetraMin® fish food (Tetra, Melle, Germany) until they reached the pupae stage.

For each batch of field-collected mosquitoes, the pupae were divided into two approximately equal-sized groups, one of which was put inside the experimental hut in the semi-field chamber, and the second group placed outside the hut. No sex separation of the mosquitoes was done, so each group of pupae yielded both male and female virgins. This experimental set-up was replicated in three different semi-field chambers, each with a similar-sized hut. The field collections were repeated weekly for 12 months, each time adding approximately half of the pupae inside and half outside the huts in each of the three chambers. Inside the experimental chambers, a 10% glucose solution soaked in cotton wool was provided as a source of energy for the emerging mosquitos. Emerged adult mosquitoes were recaptured twice weekly from inside and outside the huts (using human landing catches) as well as in the exit traps fitted to the hut windows. The recaptured female mosquitoes were kept in labeled cups and immediately assessed for insemination by dissecting and observing the spermatheca as described above. The observations were done immediately after collections in order to minimize the likelihood of mosquitoes mating inside the collection cups.

### Molecular identification of *An. funestus* sibling species

A subsample of *An. funestus* collected from the field and those emerged adults in the semi-field experiments were packed individually in microcentrifuge tubes containing silica gel. The samples were transported to the molecular laboratory at Ifakara Health Institute for sibling species identification using PCR as described by Koekemoer et al. [54].

### Statistical analyses

Data were analyzed using the open-source statistical software R version 3.6.0 [55]. Proportions and means were used for initial descriptive statistics of mosquitoes in different categories, and mean outcomes were calculated for each explanatory variable. In the first field survey, generalized linear mixed effect models (*glmer*), with a negative binomial distribution to account for overdispersion with interaction terms (house types and collection time), were used to assess mean number and variations of male mosquitoes collected inside different house types and at different collection times, i.e. early morning (7–9 a.m.), late morning (9 a.m. to 12 noon), evening (6–8 p.m.) and late evening/early nighttime (12 midnight to 2 a.m.). Random variables included in these models were the experimental round (one round involved collecting mosquitoes in each study house until collections had been performed in all study houses in each village) and household nested within villages. Additionally, pairwise comparisons were performed using Tukey’s Honestly Significant Difference test with functions provided in the *multcomp* packages in R software.

To compare the proportion of inseminated and non-inseminated mosquitoes per time, *glmer* models with binomial distribution and *logit* functions were used. For the observations of wild mosquitoes in the village, the proportion of inseminated and non-inseminated females was modeled as a function of time of mosquito sampling, i.e. evening (6 p.m.), nighttime (11 p.m.) and early morning (6 a.m.). The sampling date and hut identifier were included as random variables in the model. For observations of wild-caught mosquitoes observed in semi-field captivity, the number of inseminated and non-inseminated females was modeled as a function of the location of mosquito recapture, i.e. inside hut, outside hut or in the window exit traps. Again, the sampling date and semi-field chamber identifiers were included as random variables. Results of the models were presented as odds ratios (OR) with 95% confidence intervals (CI) and their associated* P*-values.

## Results

### Observations of male mosquitoes resting inside different types of houses

**Fig. 4:**
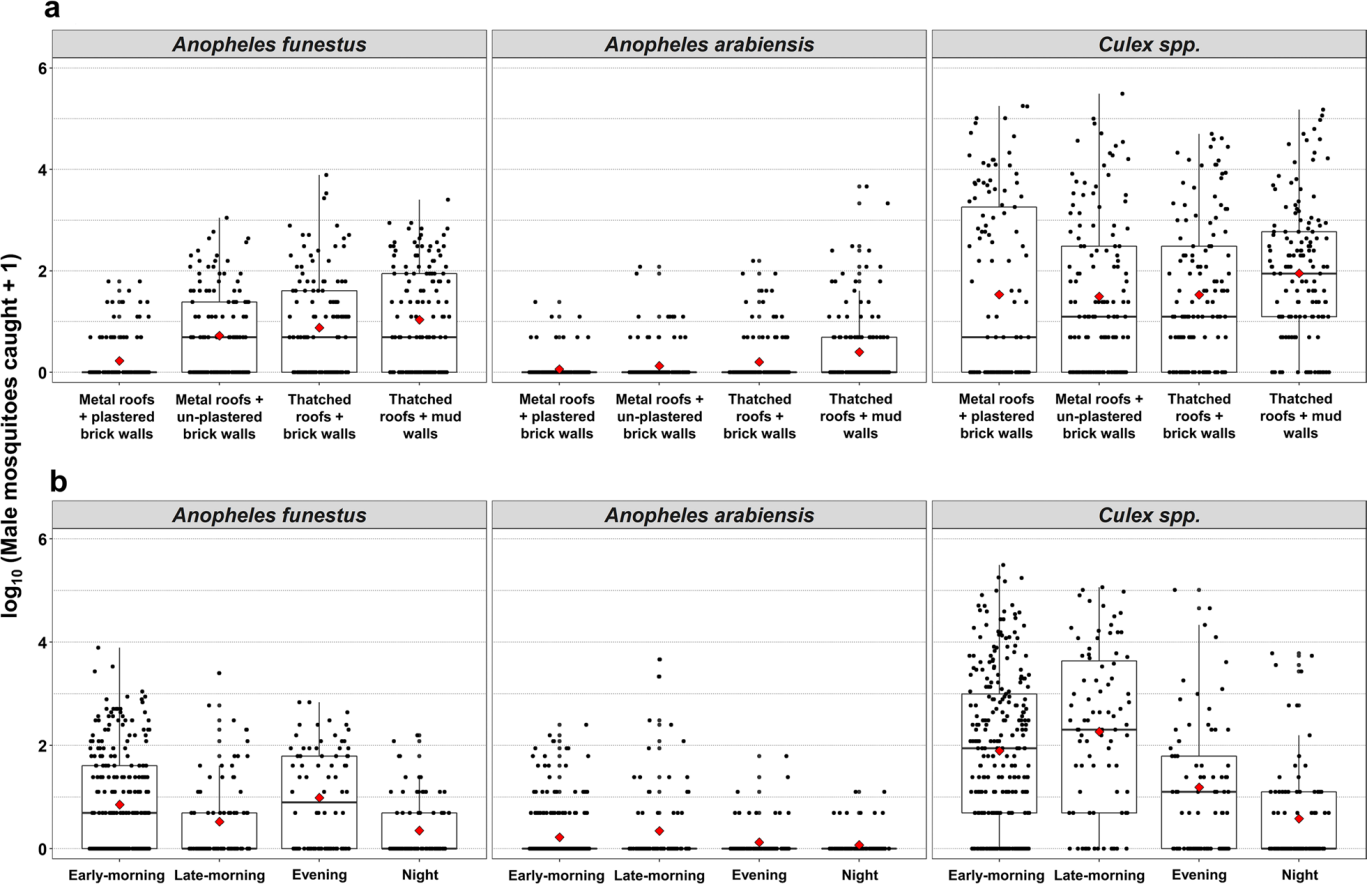
Mean differences in the densities of male mosquitoes collected from different house types (**a**) and at the different collection time points (**b**)

High densities of male mosquitoes were collected by the Prokopack trap while resting inside all house types. A total of 27,807 mosquitoes were collected indoors, comprising *An. funestus*,* An. arabiensis*,* Anopheles* coustani and *Culex* mosquitoes. Of these, 5841 were *An. funestus* (22.4% of which were males [*n* = 1306]), 1269 were *An. arabiensis* (23.0% of which were males [*n* = 292]) and 49 were *An. coustani* 49 (only 1 of which was a male mosquito); there were 20,476 *Culex* mosquitoes collected (of which 40.5% were males [*n* = 8289]). House type significantly influenced indoor densities of male mosquitoes, with thatched-roof houses having slightly higher densities of male *Anopheles* (but not *Culex*) mosquitoes than metal roof houses (Fig. 4a; Additional file 1: Table S1). The indoor densities of *Anopheles* male mosquitoes were also higher in the early- and late-morning collections than in the evening and nighttime collections (Fig. 4B; Additional file 1: Table S1).

Overall, the mean number of male *An. funestus* indoors varied from 0.14 (95% CI: 0.03–0.68) to 0.72 (95% CI: 0.16–3.26) per house per night, while the mean number of male *An. arabiensis* varied from 0.02 (95% CI: 0.01–0.13) to 0.21 (95% CI: 0.05–0.56) per house per night (Additional file 1: Table S2). The results of the analysis indicate that, although the number of mosquitoes varied with house type, the interaction between house types and time of collection showed the same trend (Additional file 1: Table S2).

### Observations of insemination in wild mosquitoes caught inside volunteer-occupied tented huts in rural Tanzania

**Fig. 5:**
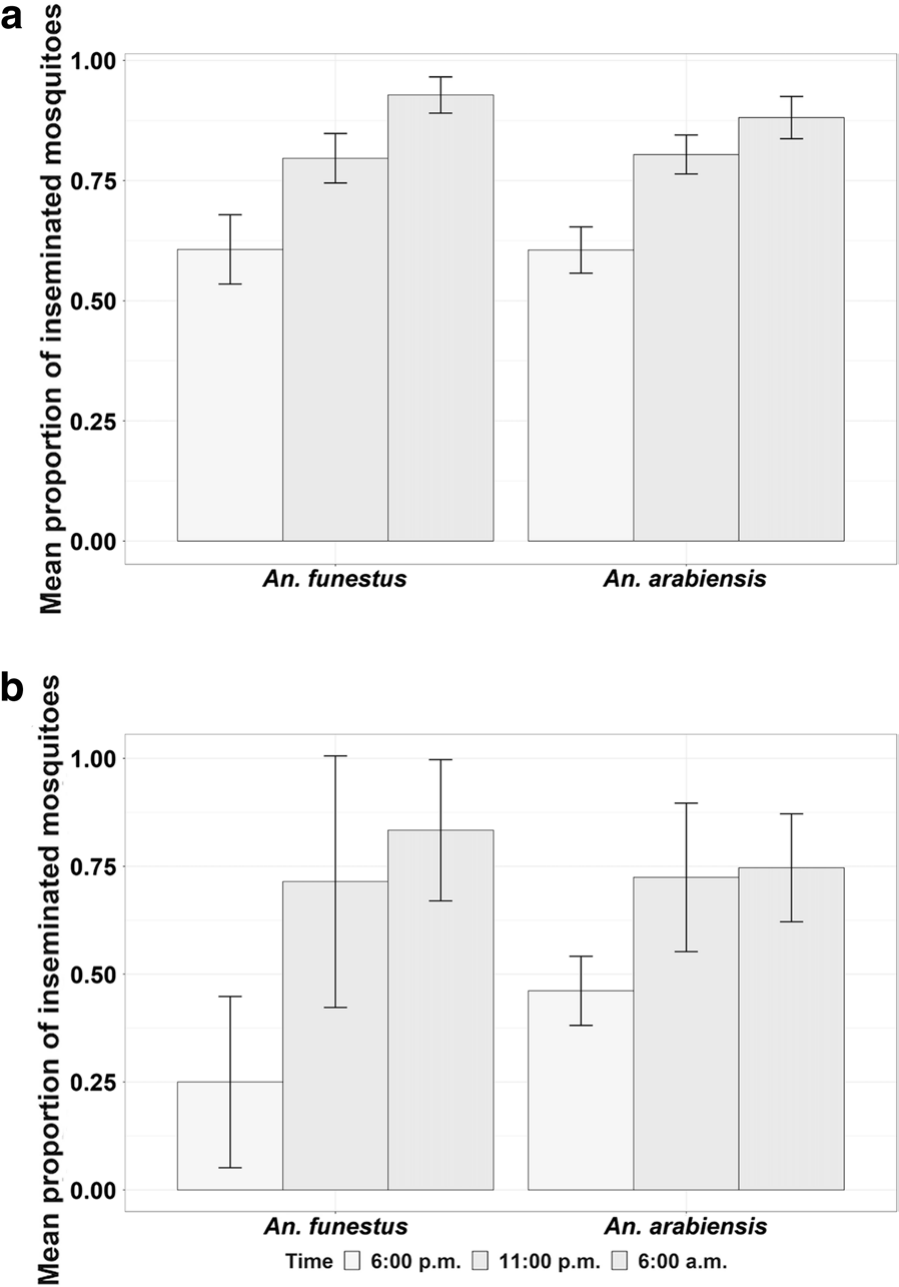
Proportions of inseminated female mosquitoes collected from the experimental huts at different time points (6 p.m., 11 p.m. and 6 a.m. the following morning). **a** Results of the first round of the experiment (14 nights) when mosquitoes were kept without freezing before dissection, **b** results of the second round of the experiment (10 nights) when mosquitoes were immobilized by freezing immediately after capture while awaiting dissection. Error bars were constructed using the 95% confidence interval of the mean

The proportion of inseminated females increased significantly after the collections at 6 p.m. and was highest in the morning collections conducted at 6 a.m., even though the huts remained completely sealed from 6 p.m. to early the following morning. A total of 594 *An. funestus* (489 females and 105 males) and 795 *An. arabiensis* (647 female and 148 male) mosquitoes were collected over the first 14 nights. The mean proportion of inseminated *An. funestus* mosquitoes increased from 60.7% (95% CI: ± 7.2%) at the 6 p.m. collections to 79.6% (95% CI: ± 5.1%) at 11 p.m. and 92.8% (95% CI: ± 3.8%) the following morning at 6 a.m. Similarly, the mean proportion of inseminated *An. arabiensis* females increased from 60.6% (95% CI: ± 4.8%) at 6 p.m. to 80.4% (95% CI: ± 4.1%) at 11 p.m. and 88.1% (95% CI: ± 4.4%) the following morning at 6 a.m. (Fig. 5a; Table 1). The *glmer* analysis showed that the increases were statistically significant (*P* < 0.001) (Table 1).

**Table 1:** Mean proportion of insemination of *Anopheles funestus* and *An. arabiensis* collected at different time points

Experiments	Collection time point	*Anopheles funestus*				*Anopheles arabiensis*			
		No. females collected	Proportion inseminated (95% CI)^a^	OR (95%CI) for insemination	*P*-value	No. females collected	Proportion inseminated (95% CI)^a^	OR (95% CI) for insemination	*P*-value
Round 1 of studies in experimental huts in rural Tanzania (14 nights)	Evening (6:00 p.m.)	191	60.7% (53.5 - 67.9)	Reference		228	60.6% (55.8 - 65.4)	Reference	
	Night (11:00 p.m.)	175	79.6% (74.5 - 84.7)	2.39 (1.50–3.79)	< 0.001	240	80.4% (76.3 - 84.5)	2.54 (1.69–3.83)	< 0.001
	Morning (6:00 a.m.)	123	92.8% (89.0 - 96.6)	6.10 (3.14–11.85)	< 0.001	179	88.1% (83.7 - 92.5)	4.91 (2.90–8.31)	< 0.001
Round 2 of studies in experimental huts in rural Tanzania (10 nights)	Evening (6:00 p.m.)	17	25% (5.2 - 44.8)	Reference		37	46.1% (38.1 - 54.1)	Reference	
	Night (11:00 p.m.)	12	71.4% (42.3 - 100)	2.28 (1.14–3.51)	0.071	36	72.4% (55.2 - 89.6)	2.46 (1.36–4.58)	0.046
	Morning (6:00 a.m.)	16	83.3% (67.0 - 99.6)	3.12 (1.26–5.42)	< 0.001	36	74.6% (62.1 - 87.1)	4.42 (2.02–6.21)	< 0.001
Studies in village houses in rural Tanzania occupied by local villagers	Evening (6:00 p.m.)	130	75.5% (67.8 - 83.2)	Reference		115	80.9% (72.8 - 89.0)	Reference	
	Night (11:00 p.m.)	107	85.9% (78.8 - 93.0)	2.09 (1.06–4.14)	0.03	128	87.6% (81.7 - 93.5)	1.63 (0.83–3.19)	0.15
	Morning (6:00 a.m.)	113	94.7% (90.7 - 98.7)	6.14 (2.56–14.74)	< 0.001	126	91.2% (86.5 - 95.9)	2.79 (1.30–6.02)	0.01

*OR* Odds ratio

^a^Confidence interval of the mean

Analysis of data from the second round of the experiment (conducted over 10 nights, during which the captured mosquitoes were immediately immobilized) revealed a similar trend of increasing proportion of insemination from 6 p.m. to 6 a.m. The mean proportion of inseminated *An. funestus* females increased from 25% (95% CI: ± 19.8%) at 6 p.m. to 71.4% (95% CI: ± 29.1%) at 11 p.m. and 83.3% (95% CI: ± 16.3%) at 6 a.m. On the other hand, the mean proportion of *An. arabiensis* increased from 46.1% (95% CI: ± 8%) at 6 p.m. to 72.4% (95% CI: ± 17.2%) at 11 p.m. and finally 74.6% (95% CI: ± 12.5%) at 6 a.m. (Fig. 5b; Table 1).

**Fig. 6:**
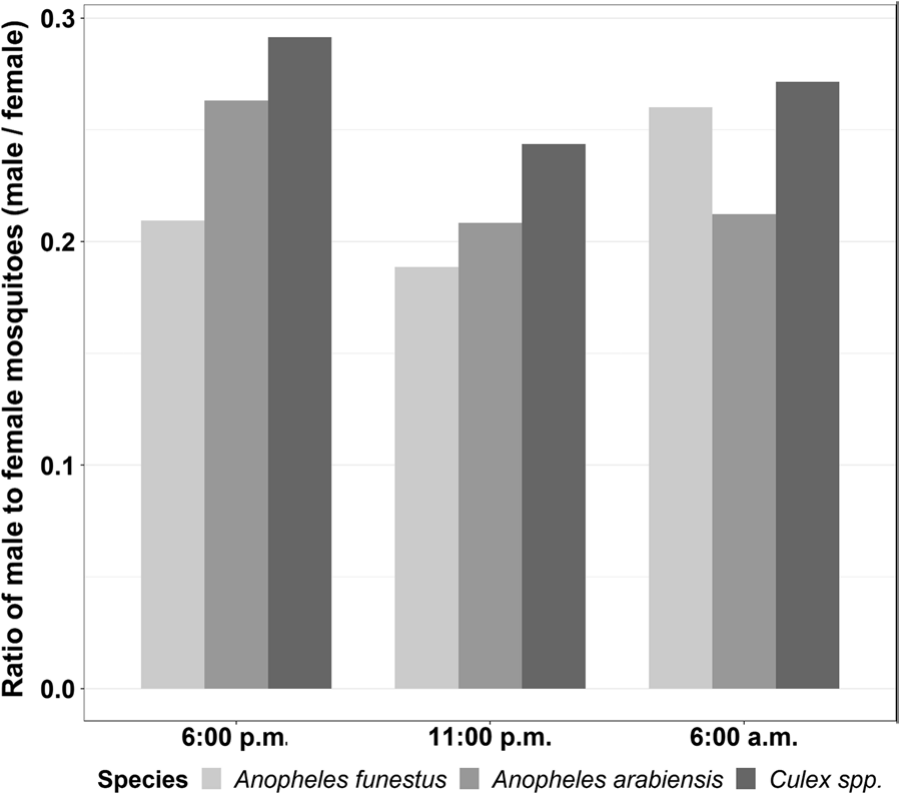
The ratio of male to female mosquitoes collected in the experimental huts calculated as the sum of males per total females collected in each house at different time points

The male to female ratio for *An. funestus* and *An. arabiensis* caught in the experimental huts at the different collection times are also shown (Fig. 6). PCR analysis showed that of all the 50 subsamples of *An. funestus* collected from the field experiment, 94% (*n* = 47) were *An. funestus* s.s. with the other 6% (*n* = 3) unamplified, while *An. arabiensis* was the only member of *An. gambiae* complex in the area.

### Observation of insemination in the wild mosquitoes caught from local houses occupied by natives in the study village

Similar to wild mosquitoes caught inside volunteer-occupied experimental huts, the proportion of inseminated females caught from houses in the study villages increased significantly after the 6 p.m. collections and was highest in the morning collections. In total, 350 female and 64 male *An. funestus* and 369 female and 74 male *An. arabiensis* were collected over 5 nights of this experiment.

**Fig. 7:**
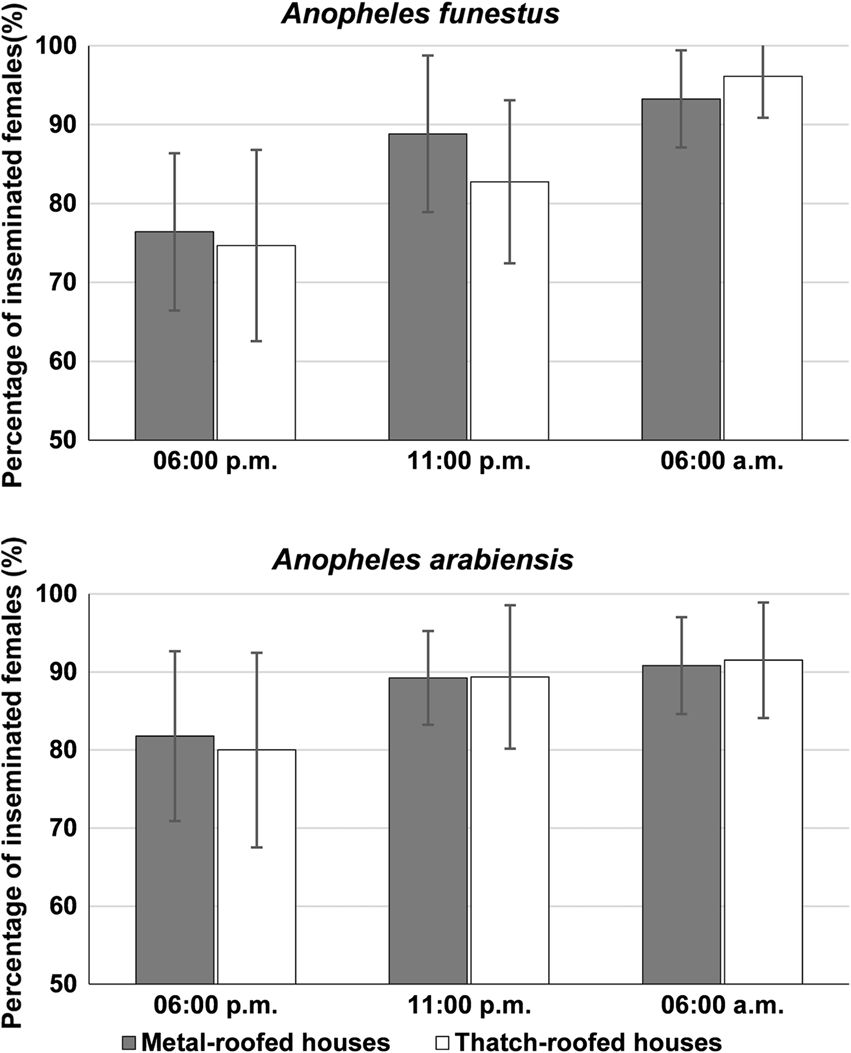
The proportion of inseminated mosquitoes collected in different house types at different time points (6 p.m., 11 p.m. and 6 a.m. the following morning). Data from village houses occupied by residents. Error bars were constructed using 95% confidence interval of the mean

Regardless of house type, the mean proportion of inseminated *An. funestus* and *An. arabiensis* females increased overnight (Fig. 7). The mean proportion of *An. funestus* increased from 76.4% (95% CI: ± 9.9%) at 6 p.m. to 88.8% (95% CI: ± 9.9%) at 11 p.m. to 93.3% (95% CI: ± 6.2%) at 6 a.m. in metal roof houses, and from 74.7% (95% CI: ± 12.1%) at 6 p.m. to 82.7% (95% CI: ± 10.3%) at 11 p.m. to 96.1% (95% CI: ± 5.3%) at 6 a.m. in thatched-roof houses. For *An. arabiensis*, the mean proportion of insemination increased from 81.8% (95% CI: ± 10.9%) at 6 p.m. to 89.2% (95% CI: ± 6.0%) at 11 p.m. to 90.8% (95% CI: ± 6.2%) at 6 a.m. in metal roof houses, and from 80% (95% CI: ± 12.5%) at 6 p.m. to 89.4% (95% CI: ± 9.2%) at 11 p.m. to 91.5% (95% CI: ± 7.4%) at 6 a.m. in thatched-roof houses (Fig. 7).

In a combined analysis of the house types, a significant increase in the proportion of inseminated female *Anopheles* mosquitoes was observed from evening to morning. However, with *An. arabiensis*, the difference in the proportion of inseminated mosquitoes collected between 6 p.m. and 11 p.m. was marginal (Table 1).

### Observations of insemination in wild-caught *Anopheles* mosquitoes maintained under semi-field conditions

In the semi-field compartments where wild-caught mosquitoes were held captive either inside or outside experimental huts (Fig. 3), a total of 3241 female *Anopheles* mosquitoes were recaptured following multiple collections over the 12 months of the experiment. Of these, 56.6% (*n* = 1833) were *An. funestus* and 43.4% (*n* = 1408) were *An. arabiensis*. Overall, 25.5% of *An. funestus* and 45.5% of *An. arabiensis* female mosquitoes that were caught were inseminated.

The mean proportions of inseminated *An. funestus* females were 16.8% (95% CI: ± 8.3%) in the exit traps, 25.2% (95% CI: ± 3.4%) inside the huts and 20.9% (95% CI: ± 2.8%) outside the huts; in comparison, the mean proportions of inseminated *An. arabiensis* females were 37.1% (95% CI: ± 6.8%) in the window exit traps, 47.4% (95% CI: ± 4.7%) inside the huts and 42.3% (95% CI: ± 5.5%) outside the huts (Table 2). Further analysis of the *An. funestus* data showed that the insemination rates were significantly higher indoors and lower in the exit traps compared to the outdoors (*P* ≤ 0.03). There was also a difference in insemination rates in *An. arabiensis* caught indoors versus outdoors although this difference was not statistically significant (*P* = 0.11). The same trend was observed for this species in samples collected from exit traps and outdoors, with the difference being marginal (*P* = 0.13) (Table 2). The proportions of inseminated *An. arabiensis* were consistently higher than those of inseminated *An. funestus* across all collection locations (outdoors, indoors and in the window exit traps) (*P* < 0.001) (Table 2).

**Table 2:** Mean proportion of inseminated *Anopheles funestus* and *Anopheles arabiensis* from the semi-field experiment

	Collection setting	*Anopheles funestus*				*Anopheles arabiensis*			
		No. females collected	Proportion inseminated (95% CI)	OR (95%CI) for insemination	*P-*value	No. females collected	Proportion inseminated (95% CI)	OR (95%CI) for insemination	*P-*value
Studies in semi-field system	Outdoor	908	20.9% (18.1 - 23.7	Ref		568	42.3% (36.8 - 47.8)	Ref	
	Indoor	792	25.2% (21.8 - 28.6)	0.56 (0.51–0.62)	0.03	568	47.4% (42.7 - 52.1)	0.55 (0.49–0.61)	0.11
	Exit trap	133	16.8% (8.5 - 25.1)	0.36 (0.26–0.48)	0.026	298	37.1% (30.3 - 43.9)	0.44 (0.37–0.52)	0.13

	*Anopheles* sp.	Indoor		Outdoor		Exit trap	
		OR (95% CI)	*P-*value	OR (95% CI)	*P-*value	OR (95% CI)	*P-*value
Interspecies comparison in a semi-field experiment	*An. funestus*	Ref		Ref		Ref	
	*An. arabiensis*	1.79 (1.33–2.41)	< 0.001	2.23 (1.62–3.08)	< 0.001	2.94 (1.77–4.90)	< 0.001

*OR* Odds ratio

PCR analysis of the 905 emerged adult *An. funestus* in the semi-field study revealed that 84.9% (*n* = 769) were *An. funestus* s.s., 6.5% (*n* = 59) were *Anopheles rivulorum*, 0.9% (*n* = 8) were *Anopheles leesoni* and the remaining 7.6% (*n* = 69) were unamplified. While most of the mosquitoes mating indoors and outdoors were *An. funestus*s.s. or *An. arabiensis*, there were also *An. rivulorum* mosquitoes that mated both indoors and outdoors.

## Discussion

Mating in mosquitoes is considered to occur as in many other dipterans, mostly in swarms in different arenas [30], but it is recognized that mating can also occur independently of swarms [39]. The focus of this study was to investigate the indoor and outdoor mating successes of the main malaria vectors in rural Tanzania, *An. funestus* and *An. arabiensis*. The study used a four-phase approach consisting of: (i) assessment of indoor resting densities of male mosquitoes inside human dwellings in rural villages; (ii) field observations of wild mosquitoes entering huts occupied by volunteers that had been constructed in the same villages; (iii) field observation of insemination status of wild mosquitoes trapped from natural houses in the village; and (iv) semi-field observations of wild-caught mosquitoes to verify and quantify insemination indoors and outdoors.

The three main findings were as follows: (i) a significant number of male mosquitoes rest indoors in different house types and the densities of these male mosquitoes are highest during the morning; (ii) approximately 60% of female *Anopheles* mosquitoes collected indoors were already inseminated at 6 p.m., but this proportion increased to 90% by the following morning even when the huts remained closed after the 6.p.m collection, implying additional mating indoors despite no additional mosquito entry; and (iii) under semi-field settings, wild-caught *Anopheles* mosquitoes held inside huts mated as frequently as those outdoors.

These observations confirm that while most mating in the wild may be happening outdoors, there is substantial additional mating that can occur after the mosquitoes are already indoors. Both *An. funestus* and *An. arabiensis* show this flexibility in behaviour although indoor insemination appeared to be more prominent in *An. funestus* for which indoor insemination exceeded that outdoors. The experimental hut studies conducted by Dao et al. [39] in West Africa found that mating in mosquitoes exiting huts was 5% higher than that in those entering, thus providing the first indications of this phenomenon in malaria mosquitoes. Our study supports that original hypothesis but also demonstrates that indoor mating can be far more substantial. Moreover, unlike the outdoor mating in swarms, which typically happens at dusk [33, 36, 56, 57], the present study shows that indoor mating can happen far later in the evening and also during the night. Our field study showed a gradual increase in insemination from approximately 60% at 6 p.m. to 80% at 11 p.m. and finally to 90% at 6 a.m. the following morning, suggesting continued mating events throughout the night. Since no observations were made of any swarms indoors, it is unclear whether the indoor mating is a function of swarms or otherwise.

This study also highlights the influence of different house types on indoor resting densities of male mosquitoes. Males of both *An. funestus* and *An. arabiensis* showed a preference for less improved houses, such as those with thatched roofs, compared to more improved houses, such as those with metal roofs. Similar preferences have been demonstrated in *Anopheles* females in several studies [49, 58, 59]. One clear implication of this observation is that indoor interventions that typically target host-seeking female mosquitoes, such as indoor residual spraying (IRS) [15], may also impact males, possibly explaining why IRS campaigns have been particularly effective against *An. funestus* populations, for which substantial densities of males rest indoors [60, 61]. Fortunately, insecticide resistance surveys in rural Tanzania have shown that *Anopheles* males have a similar phenotypic expression of resistance as their conspecific females, and would therefore respond similarly to non-pyrethroid IRS treatments [62]. Similarly, some of the new vector control tools being evaluated to complement ITNs and IRS, notably attractive toxic sugar baits [26, 28, 63], could be optimized to target male and female mosquitoes indoors.

An important question is whether the experimental set-up used in this study induced the observed indoor insemination or whether this was entirely a natural phenomenon. While it is impossible to rule out an impact of the experimental set-up, the actual findings suggest that indoor mating is a natural phenomenon, albeit occurring at a lower frequency than outdoor mating. The natural observation of males indoors suggests that there are several opportunities for the female and male mosquitoes to copulate. Moreover, the experimental huts used in the semi-field observations were unnatural and fitted with window exit traps so that flying mosquitoes could exit but not enter the huts. Yet there was still substantial insemination indoors, with the mean proportions being equal to or higher than those observed outdoors or in exit traps. It can be inferred, therefore, that both *An. funestus* and *An. arabiensis* adults could voluntarily mate indoors despite the unrestricted egress routes. This inference was confirmed by the observed indoor insemination in local village houses, which remained unsealed during the experiment. Future studies should examine the proportions of virgin females at different time points during the night, and the potential effect of egressing virgins on the proportion of residual female indoors that are inseminated.

All of the field studies were completed on a nightly basis and the huts cleaned thoroughly each day. The field observations cannot therefore be used to infer the true quantities of indoor mating in nature but are merely an indication that this phenomenon does indeed happen. Interestingly, however, most of the mosquitoes that entered the huts had already mated. As can be seen in the field data, approximately two thirds of the *Anopheles* females were already inseminated when they entered the huts. Most likely these females had already left the swarm stations. We found a small but consistent additional mating of about 30% in the first round in the experimental huts and approximately 19% in the local dwellings. It would appear that when confined, and in the absence of any additional recruitment of males or females, the actual proportions of inseminated mosquitoes will be high even if any additional insemination indoors is minimal. Another question is whether swarming was actually necessary for such indoor mating. We did not observe any evidence of swarming indoors; thus, the indoor mating observed in our study appeared to be happening without any apparent swarming. However, our findings must be considered to be preliminary, and it is important that this specific question be evaluated in future research.

Another limitation of this study was that neither the gonotrophic state of the females nor the status of the genitalia of the males were observed. Direct observations of the rotation of the male genitalia to assess sexual maturity or their antennas to assess readiness for mating could improve the degree of certainty for the indoor mating phenomena. It will be interesting to assess changes in gonotrophic state of the female, how this changes overnight and whether there are any correlations between this and the insemination rates. It is also possible that the trapping methods used, in particular the human landing catches, may have introduced some biases, although these do not invalidate the overall findings.

To accelerate efforts towards malaria elimination, the deployment of novel approaches to complement current vector control (ITNs and IRS) are essential [16]. Such tools should particularly mitigate current challenges, such as insecticide resistance to the commonly used pyrethroids [64–67] and outdoor biting by malaria vectors [68–73]. The findings of this study on mating behavior, among other aspects of the *Anopheles* life-cycle processes, may provide insights towards the development or improvement of malaria vector control. The results may be used to design or improve indoor insecticidal methods targeting males, females or both, as well as niche products such as ATSBs to also target male and female mosquitoes indoors and outdoors.

Another potential opportunity from these findings is in the area of mosquito colonization inside laboratories, which is currently a challenge for some vector species. In particular, *An. funestus*, which mediates significant proportions of ongoing malaria transmission, particularly in southern and East Africa, remains one of the most difficult mosquito species to rear inside laboratories. Laboratory colonies are an essential requirement for experimental studies under controlled conditions, such as enabling characterization of insecticide resistance [62, 74, 75] as well as studies on other genetic traits [76, 77], immune strategies [78, 79] and key vector demographic profiles [80–82]. Unlike *An. gambiae*, *An. funestus* remains extremely difficult to colonize and maintain inside laboratories, partly because it is eurygamic (does not mate in captivity) and has poorly understood ecological needs. To our knowledge, only two strains have been successfully colonized from wild populations despite several attempts, both at the Vector Control Reference Laboratory (VCRL) in the National Institute for Communicable Diseases, South Africa, from populations collected in Angola (FANG) and Mozambique (FUMOZ) [44]. The FUMOZ strain is also maintained at other laboratories worldwide, including in Cameroon, UK [75] and Tanzania [45].

The field observations by Dao et al. focused on *An. coluzzii* and demonstrated that under confinement there was a breakdown of the cross-species mating barrier when other species were added indoors in experimental houses [39]. This suggests that indoor conditions are not sufficiently representative of actual mating conditions in the wild, but that the same conditions could be favorable for breaking the bottlenecks associated with mating in some species, such as *An. funestus*, which have been challenging to rear inside laboratories, partly because of poor mating. The present study improves upon the experiments by Dao et al. [39] by introducing controlled observations in both the semi-field and field settings and validating the observations underconditions of both restricted and unrestricted egress.

Several attempts are being made to colonize new *An. funestus* strains [83] from wild populations, but methods used to establish FUMOZ and FANG have not been successful elsewhere [84], including those attempted in the same wild populations from which FUMOZ was originally derived (M Coetzee, personal communication). The inability to repeatedly colonize and establish *An. funestus* in laboratories is responsible for the more limited understanding of the biology of this species compared to other vector species. Recent evidence from colonization attempts in Tanzania has highlighted mating as one of the bottlenecks to colonization [45]. The findings in the present study may therefore enable advancement towards alternative colonization processes either inside laboratories or by using semi-field chambers and experimental huts in which natural mating can happen.

## Conclusion

This study demonstrates that wild populations of *An. funestus* and *An. arabiensis* can mate both inside and outside dwellings. Most mating likely happens outdoors before the mosquitoes enter houses, but significant additional mating can happen indoors. The indoor insemination in huts with exit traps indicates that mosquitoes can voluntarily mate indoors despite unrestricted egress. These findings may be relevant for improving vector control by targeting male mosquitoes and may also inform improved efforts to colonize *Anopheles* species inside laboratories or semi-field chambers.

## Supplementary Information

**Additional file 1.** Male mosquitoes in different house categories and collection time.

## Data Availability

All data generated from this study will be available from the corresponding authors upon request.

## Abbreviations

CI: Confidence interval
GMM: Genetically modified mosquitoes
ITNs: Insecticide-treated nets
IRS: Indoor residual spraying
SIT: Sterile insect techniques
PCR: Polymerase chain reaction
