# Supplementary material for: Wild populations of malaria vectors can mate both inside and outside human dwellings
Source: Parasit Vectors. 2021 Oct 7;14:514. doi: 10.1186/s13071-021-04989-8 (PMC8499572; doi:10.1186/s13071-021-04989-8)
Supplement: Supplementary file 1 — Additional file 1. Male mosquitoes in different house categories and collection time. [file 13071_2021_4989_MOESM1_ESM.docx]

**Additional file 1: Table S1**

Summary statistics for pairwise comparison of number of male mosquitoes of different species collected in different house types and different collection times.

|  | *Anopheles funestus* | | *Anopheles arabiensis* | | *Culex mosquitoes* | |
| --- | --- | --- | --- | --- | --- | --- |
|  | | | | | | |
| Comparison | Mean difference  (95% CI) | p value | Mean difference (95% CI) | p value | Mean difference  (95% CI) | p value |
| **House type** | | | | | | |
| Metal roofs and plastered brick walls - Thatched roofs and brick walls | -1.77 (-2.25 - -1.29) | 0.001 | -1.45 (-2.12 - -0.78) | 0.13 | -0.07 (-0.46 - 0.32) | 0.998 |
| Metal roofs and un-plastered brick walls - Thatched roofs and brick walls | -0.55 (-1.00 - -0.10) | 0.60 | -0.22 (-0.78 - 0.34) | 0.98 | 0.42 (0.04 - 0.80) | 0.68 |
| Thatched roofs and mud walls - Thatched roofs and brick walls | -0.31 (-0.75 - 0.13) | 0.90 | 0.48 (-0.06 - 1.02) | 0.8 | 0.29 (-0.10 - 0.68) | 0.87 |
| Metal roofs and un-plastered brick walls - Metal roofs and plastered brick walls | 1.21 (0.73 - 1.69) | 0.05 | 1.23 (0.57 - 1.89) | 0.24 | 0.49 (0.11 - 0.87) | 0.56 |
| Thatched roofs and mud walls - Metal roofs and plastered brick walls | 1.46 (0.99 - 1.93) | 0.01 | 1.95 (1.31 - 2.59) | 0.01 | 0.36 (-0.02 - 0.74) | 0.77 |
| Thatched roofs and mud walls - Metal roofs and un-plastered brick walls | 0.24 (-0.20 - 0.68) | 0.95 | 0.7 (0.17 - 1.23) | 0.55 | -0.13 (-0.50 - 0.24) | 0.986 |
|  | | | | | | |
| **Collection time** | | | | | | |
| Evening - Early morning | -0.33 (-0.51 - -0.15) | 0.25 | -0.69 (-0.99 - -0.39) | 0.09 | -0.18 (-0.37 - 0.002) | 0.74 |
| Late morning - Early morning | 0.09 (-0.15 - 0.33) | 0.98 | -0.05 (-0.32 - 0.22) | 0.998 | 0.11 (-0.06 - 0.28) | 0.90 |
| Mid night - Early morning | -1.69 (-1.90 - -1.48) | <0.001 | -1.47 (-1.82 - -1.12) | <0.001 | -1.29 (-1.48 - -1.10) | <0.001 |
| Late morning - Evening | 0.41 (0.11 - 0.71) | 0.5 | 0.65 (0.24 - 1.06) | 0.37 | 0.3 (0.05 - 0.55) | 0.61 |
| Mid night - Evening | -1.36 (-1.58 - -1.14) | <0.001 | -0.77 (-1.18 - -0.36) | 0.22 | -1.1 (-1.31 - -0.89) | <0.001 |
| Mid night - Late morning | -1.78 (-2.10 - -1.46) | <0.001 | -1.42 (-1.86 - -0.98) | 0.01 | -1.4 (-1.65 - -1.15) | <0.001 |

**Additional file 1: Table S2**

Showing model estimated means of male *Anopheles* mosquitoes collected from different house types and different collection time points

|  | | *Anopheles funestus* | | *Anopheles arabiensis* | | *Culex mosquitoes* | |
| --- | --- | --- | --- | --- | --- | --- | --- |
|  | | | | | | | |
|  | Number of collections | Number of mosquitoes | Mean (95% CI) | Number of mosquitoes | Mean (95% CI) | Number of mosquitoes | Mean (95% CI) |
| **House type** | | | | | |  |  |
| Metal roofs and plastered brick walls | 121 | 54 | 0.14 (0.03-0.68) | 13 | 0.02 (0.01-0.13) | 2385 | 7.52 (2.16-26.21) |
| Metal roofs and un-plastered brick walls | 134 | 285 | 0.43 (0.09-2.00) | 35 | 0.12 (0.05-0.27) | 1981 | 12.34 (3.55-42.90) |
| Thatched roofs and brick walls | 128 | 428 | 0.72 (0.16-3.26) | 58 | 0.13 (0.04-0.37) | 1614 | 8.08 (2.33-27.97) |
| Thatched roofs and mud walls | 143 | 539 | 0.43 (0.09-2.06) | 186 | 0.21 (0.05-0.56) | 2358 | 10.84 (3.10-37.92) |
|  | | | | | |  |  |
| **Collection time** | | | | | |  |  |
| Early-morning | 273 | 851 | 0.36 (0.08-1.49) | 136 | 0.10 (0.04-0.26) | 5101 | 9.47 (3.09-29.07) |
| Late-morning | 87 | 142 | 0.37 (0.09-1.62) | 125 | 0.09 (0.03-0.28) | 2278 | 10.62 (3.38-33.36) |
| Evening | 78 | 248 | 0.28 (0.06-1.14) | 18 | 0.05 (0.02-0.15) | 671 | 7.89 (2.44-25.48) |
| Mid night | 86 | 65 | 0.08 (0.01-0.27) | 10 | 0.02 (0.01-0.10) | 239 | 2.61 (0.80-8.49) |
|  |  |  |  |  |  |  |  |
| **Interaction (House type and collection time)** |  |  |  |  |  |  |  |
| Metal roofs and plastered brick walls:Morning | - | - | 0.88(0.20-3.86) | - | 1.13 (0.9-1.36) | - | 1.83(0.70-4.77) |
| Metal roofs and unplastered brick walls:Morning | - | - | 0.89(0.20-3.93) | - | 0.10 (0.04-0.29) | - | 2.08(0.76-5.75) |
| Thatched roofs and mud walls:Morning | - | - | 1.23(0.32-4.71) | - | 0.98 (0.4-1.28) | - | 1.84(0.74-4.56) |
| Metal roofs and plastered brick walls:Evening | - | - | 0.13(0.02-1.27) | - | 0.2 (0.01-0.16) | - | 1.61(0.56-4.65) |
| Metal roofs and unplastered brick walls:Evening | - |  | 1.29(0.53-3.18) | - | 0.3(0.01-0.63) | - | 1.35(0.49-3.73) |
| Thatched roofs and mud walls:Evening | - | - | 0.97(0.42-2.23) | - | 0.3 (0.01-0.72) | - | 0.93(0.39-2.19) |
| Metal roofs and plastered brick walls:Mid night | - | - | 2.52(0.49-12.91) | - | 0.20 (0.05-0.41) | - | 2.42(0.74-7.91) |
| Metal roofs and unplastered brick walls:Mid night | - | - | 4.22(1.42-12.54) | - | 0.11 (0.03-0.33) | - | 2.62(0.84-8.17) |
| Thatched roofs and mud walls:Mid night | - | - | 0.99(0.33-2.94) | - | 0.20 (0.06-0.49) | - | 0.93(0.34-2.54) |

Means were calculated from *glmer* models
